# Map Sensitivity vs. Map Dependency: A Case Study of Subway Maps’ Impact on Passenger Route Choices in Washington DC

**DOI:** 10.3390/bs7040072

**Published:** 2017-10-25

**Authors:** John Xu

**Affiliations:** Department of Science, Deerfield Academy, Deerfield, MA 01342, USA; johnnyxu999@gmail.com; Tel.: +1-617-838-6776

**Keywords:** route choice, schematic map, frequent rider, behavior, Washington DC, prospect theory, Mechanical Turk

## Abstract

This paper addresses the key assumption in behavioral and transportation planning literature that, when people use a transit system more frequently, they become less dependent on and less sensitive to transit maps in their decision-making. Therefore, according to this assumption, map changes are much less impactful to travel decisions of frequent riders than to that of first-time or new passengers. This assumption—though never empirically validated—has been the major hurdle for transit maps to becoming a planning tool to change passengers’ behavior. This paper examines this assumption using the Washington DC metro map as a case study by conducting a route choice experiment between 30 Origin-Destination (O-D) pairs on seven metro map designs. The experiment targets two types of passengers: frequent metro riders through advertisements on a free daily newspaper available at DC metro stations, and general residents in the Washington metropolitan area through Amazon Mechanical Turk, an online crowdsourcing platform. A total of 255 and 371 participants made 2024 and 2960 route choices in the respective experiments. The results show that frequent passengers are in fact more sensitive to subtle *changes* in map design than general residents who are less likely to be familiar with the metro map and therefore unaffected by map changes presented in the alternative designs. The work disproves the aforementioned assumption and further validates metro maps as an effective planning tool in transit systems.

## 1. Introduction

Traditional economic theory makes the assumption that people solve problems and make choices entirely rationally. These assumptions lead to the expectation that people behave like the *Homo economicus*, basing every decision solely on utility maximizations. However, human behavior suggests that we are more straightforward with our choices. Bounded rationality, proposed by Simon, suggests that we are inclined to make decisions by “satisficing” [1]. Most of the time, people’s judgments are suboptimal [2]. A good example of suboptimal judgment is how metro route choices are made, as passengers often lean away from harder-to-obtain perfect travel information and opt for readily accessible imperfect information present at metro stations.

Metro transit has become an increasingly popular means with which people travel between places. Transit users rely heavily on information presented to them during planning, weighing in factors such as hours, fares, transfers, waiting times, station availability, boarding locations, and vehicle identification [3,4]. When trying to juggle between these factors, travelers often find themselves presented with more than one reasonable route between given origins and destinations. 

Traditional beliefs in transportation planning make two key assumptions. First, it is assumed that travelers would choose the route that optimizes travel time and cost. However, travelers in real-time consider a variety of other factors, with schematic maps contributing as the single most important resource in providing crucial yet imperfect visual and spatial information. With many aesthetic and geographical considerations packed into one frame, transit maps readily induce the non-*Homo economicus* rider to become dependent on the map’s generalizations and to consistently choose “satisficing”—not necessarily optimized—routes. This paper explores the ways in which schematic transit maps can affect travel behavior and confirms several possibilities of doing so within the Washington DC metro system. 

The second assumption, on the other hand, has long hindered transit maps from becoming an effective planning tool. The belief is considered a “reasonable extension” of the research observation that, as a passenger becomes more familiar with a transit system, he or she would be more likely to rely on his or her experience rather than publicly-displayed schematic maps. Lotan [5] found that familiar and new users could react differently to transit information, while Emmerink [6] established that frequent riders are more likely to rely on their experience when navigating transit systems. 

In assuming that frequent riders are both less dependent on *and* not sensitive to maps when traveling, planners have all but nullified the possibility of using transit maps to mitigate crowding problems during peak hours. This paper conducts one of the first experiments on the aforementioned unproven yet widely accepted belief on map sensitivity. The tests show a strong pattern that contradicts this conventional belief, suggesting that although frequent riders are not always map-dependent, they are more map-sensitive than infrequent riders and are often more susceptible to map visualization changes. 

By comparing traveler route choices from seven different maps, this paper calls to attention the possibility of using humans’ bounded rationality to redirect traffic and to alleviate transit capacity constraints. Redrawing transit maps is a low cost and low public disapproval alternative to other traditional methods taken to mitigate congestion, such as service changes or building infrastructure. Accounting for the route preferences of several ridership groups in the DC metro with different familiarity levels, this paper introduces to a limited literature a new perspective on maps’ potential impact on ridership behavior. 

## 2. Literature

Relevant literature in marketing, economics, cartography, and psychology emphasizes the importance of visualization for consumers with different product familiarity levels. Berkowitz [7] found product form to be the basis for inferences about other key product attributes. Wansink, van Ittersum, and Painter [8] discovered that, at an ice cream social, 34 ounce bowls induced a higher per capita ice cream consumption than 17 ounce bowls. Elsewhere, even though literature is yet to produce a conclusive set of guidelines for schematic map design, Bailenson, Shum, and Uttal [9,10] found that travelers prefer routes that start straight, while Dalton [11] further observed their tendency to preserve general linearity. Gollege and Garling [12] set forth an inconclusive but more comprehensive list of route selection criteria. 

The impact of visualization on traveler behavior has been analyzed in a small but emerging body of literature. A classic example is Lake Shore Drive, Chicago [13], where the number of accidents at a dangerous turn decreased by 36% with a simple length misconception: by painting decreasingly spaced lines perpendicular to the direction of travel leading up to the curve, transportation authorities gave drivers the perception that they were speeding up, causing them to hit the brakes earlier. 

In the field of schematic transit maps, Guo [14] found that the London tube map has a large impact on riders’ route choice decisions: passengers trust the information shown on the map twice as much as information on the actual traveling time. In this work, Guo found that, as measured by elasticity, frequent riders are 21% less sensitive to the current map than novice riders. However, Guo did not present any alternative maps and only tested the effect of the existing tube map. Raveau, Muñoz, and De Grange [15] and Raveau, Guo, Muñoz, and Wilson [16] followed a similar approach in analyzing the metro system in Santiago, Chile, based on survey data from 28,961 passengers on 1365 Origin-Destination (O-D) pairs. Although they discovered that maps matter to passengers’ route choices, they did not present alternative designs to the current map, and the study was thus unable to answer the important question of how familiar passengers would change their behavior when shown different maps. 

Two works investigated alternative maps in a lab setting. Roberts et al. [17] asked participants to plan their trips between five O-D pairs on three distinct map designs in Paris, France. Though the alternative maps follow different design principles (curved, instead of traditional octolinear), all participants were unfamiliar with the current official map. Thus, Roberts was unable to detect frequent riders’ sensitiveness to changes on the existing map. Hochmair [18] investigated route choices of 35, primarily undergraduate, participants on four different maps of the Vienna subway. However, Hochmair’s alternative maps only added new annotations and did not change the fundamental design of the current map. 

One of the most sophisticated works to date was done by Guo et al. [19] using Amazon Mechanical Turk. Guo asked 2655 participants to choose their routes between eight Origin-Destination pairs on one of seven maps of the DC subway. Although answering key questions on general passenger behavior, Guo collected results from an overwhelmingly novice participant base (given Mechanical Turk’s general representativeness of the population) and thus did not focus on differences in behavior between frequent and infrequent riders when shown alternative designs. 

This research follows a similar approach as Guo et al. [19]. Using Guo’s existing alternative maps and O-D pairs, it conducts the experiment on two distinct participant groups: those frequent riders who are very familiar with the Washington DC subway, and those infrequent riders who are less familiar with the system. With the results showing frequent riders to be more sensitive to changes on alternative maps, this work legitimizes map design as a powerful tool to shift passenger behavior and alleviate transit systems’ capacity constraints. 

## 3. Congestion in the DC Subway

The Washington DC metro system, operated by the Washington Metropolitan Area Transit Authority (WMATA, Washington, D.C., United States), includes six lines and 91 stations covering 117 miles. It is the second largest subway system in the United States by ridership, serving not only the District of Columbia, but also surrounding counties in Maryland and Virginia. 

The DC metro system is, at each track, capable of handling 26 trains per hour (TPH), or approximately one train per 135 seconds (WMATA *PlanItMetro*). The metro crosses the Potomac River twice from Virginia into DC: the Rosslyn Tunnel is shared by the Orange, Blue, and Silver Lines, while the 14th Street Bridge is used by the Yellow Line. The Rosslyn Tunnel has been the chokepoint of the entire metro system. In as early as 2012, before the construction of Silver Line Phase I, the Rosslyn Tunnel already processed trains at full capacity. Its 26 TPH consisted of 16 Orange Line and 10 Blue Line trains. The Yellow Line’s crossing, 14th Street Bridge, however, was running at 38% capacity with 10 TPH. The Yellow Line cannot operate at more than 14 TPH, or 54% capacity, as it shares track with the 12 TPH Green Line at stations due north of L’Enfant Plaza (Greater Greater Washington). Thus, in 2012, a total of 36 TPH was entering DC from Virginia by crossing the Potomac River. 

**Figure 1:**
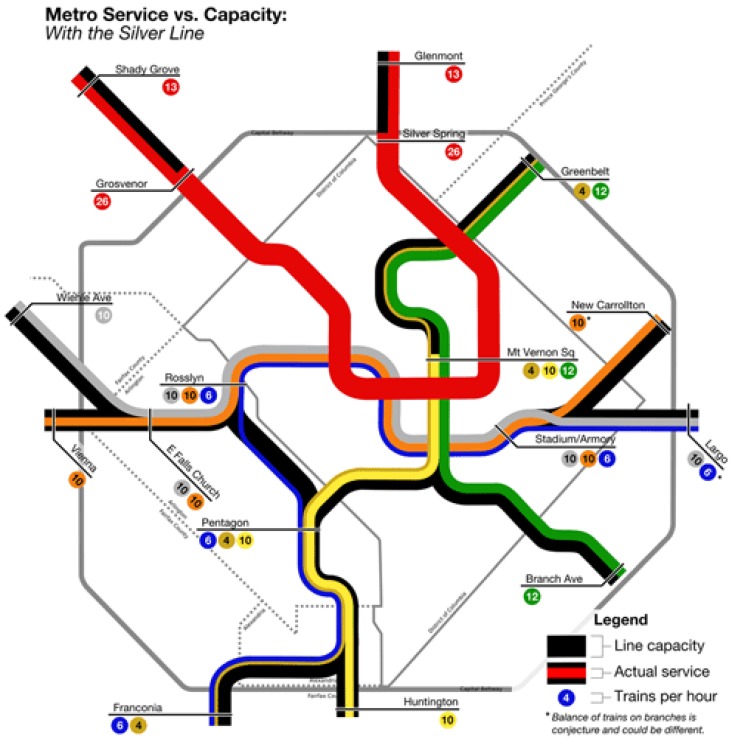
Metro Service vs. Capacity (*Greater Greater Washington*).

When the Silver Line Phase I opened in July 2014, WMATA removed trains from both the Orange and the Blue Line to allow Silver Line trains to share the track, adding some additional capacity to the Yellow Line with the hope of utilizing the underused crossing at the 14th Street Bridge (Figure 1). However, passengers did not follow the service change as expected. The reduced-capacity Blue Line became more congested and trains through stations near Rosslyn Tunnel became the most crowded in the system, often reaching crashing level with more than 120 passengers per car (WMATA *Vital Signs Report*, Q3 2015). To make matters worse, due to overcrowding, congestion, and delay, the Rosslyn Tunnel can no longer process 26 TPH; WMATA now officially runs Blue Line trains at 5 TPH instead of the planned 6 TPH (WMATA.com). In the mean time, the Yellow Line remains underused with high levels of spare capacity and relatively empty platforms even during peak hours. With fewer trains available, the waiting times for Blue Line riders has increased significantly over the last three years, from 6 min per train (10 TPH) to 12 min per train (5 TPH). 

In using a very traditional method, WMATA shifted down the supply of Blue Line trains and up that of the Yellow Line, hoping that demand would follow. Yet as riders neither take the pains to optimize nor behave as *Homo economicus* would, WMATA’s efforts have been quite unsuccessful. This paper explores the possibility and potential impact of an alternative solution: to shift behavior by changing the relative lengths of the Yellow and Blue Lines (by shortening the Yellow Line or elongating the Blue Line) on the metro map. 

## 4. Proposal and Methodology

### 4.1. Alternative Map Designs

**Figure 2:**
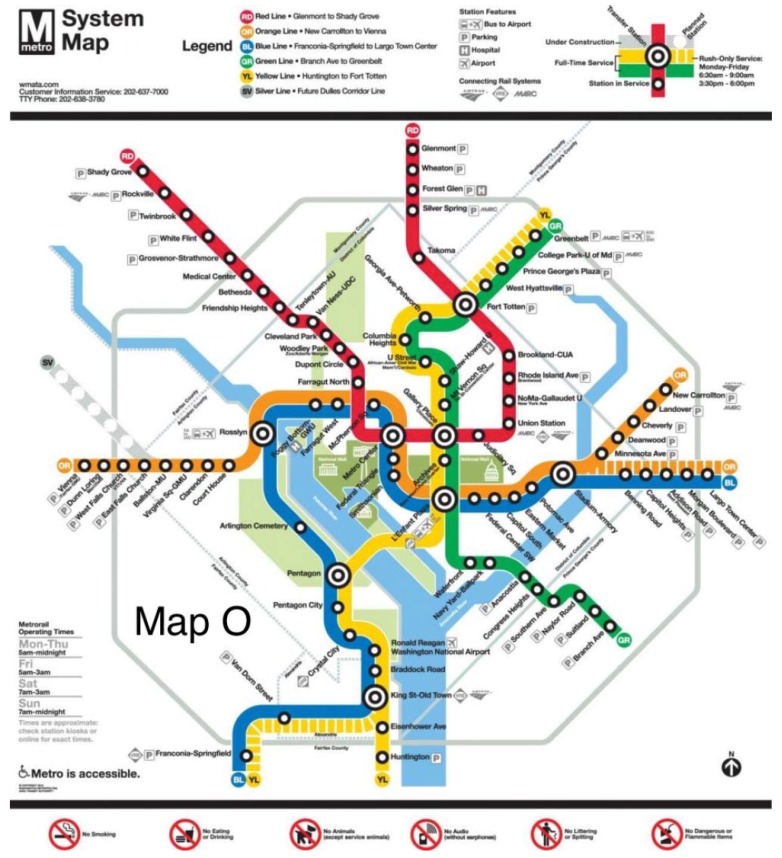
Official DC metro system map with Silver Line under construction, 2014 (WMATA).

**Figure 3:**
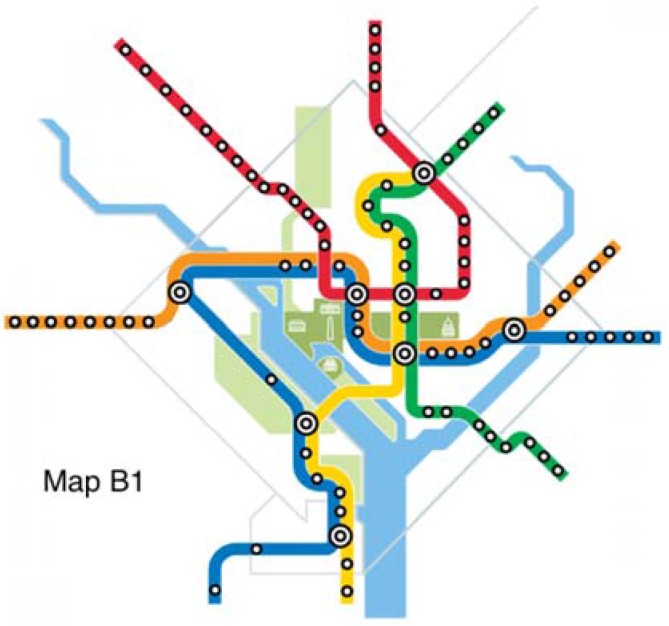
Map B1.

**Figure 4:**
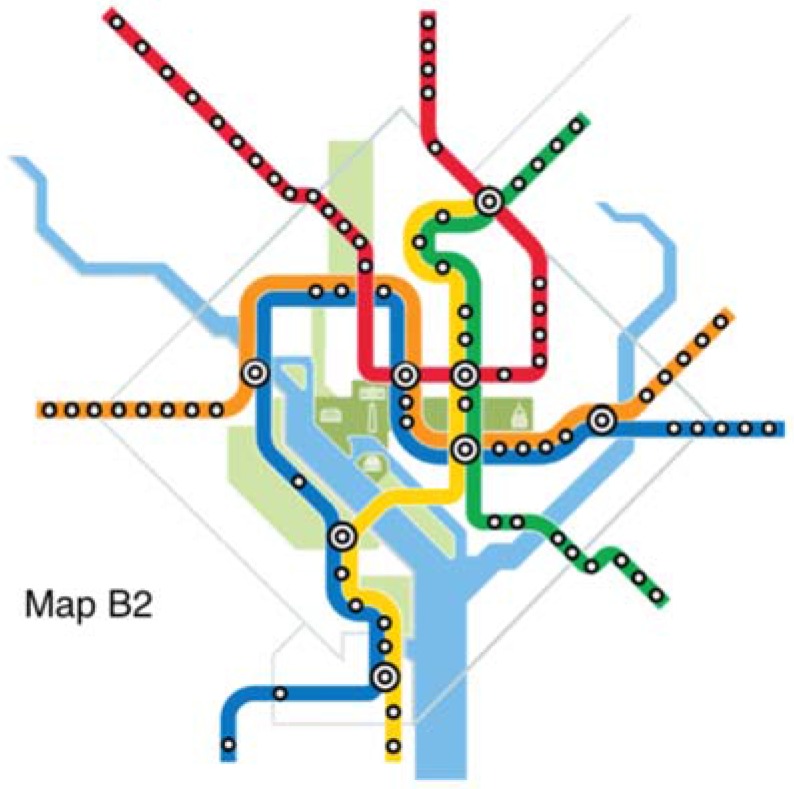
Map B2.

**Figure 5:**
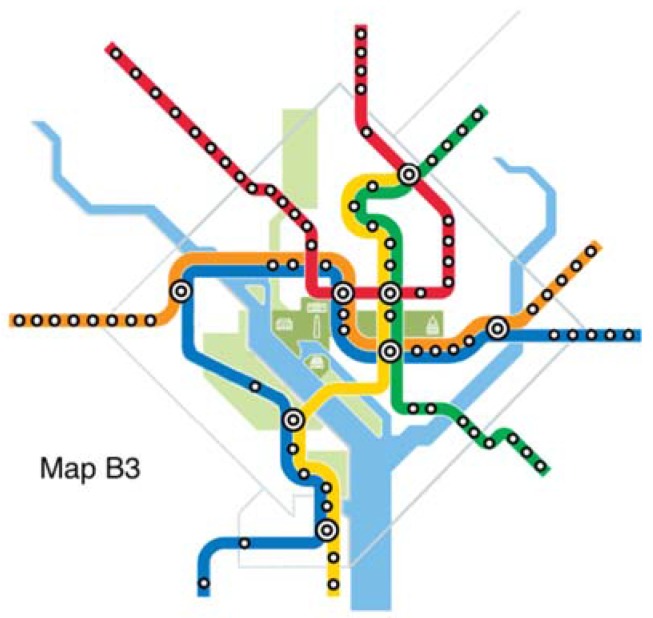
Map B3.

**Figure 6:**
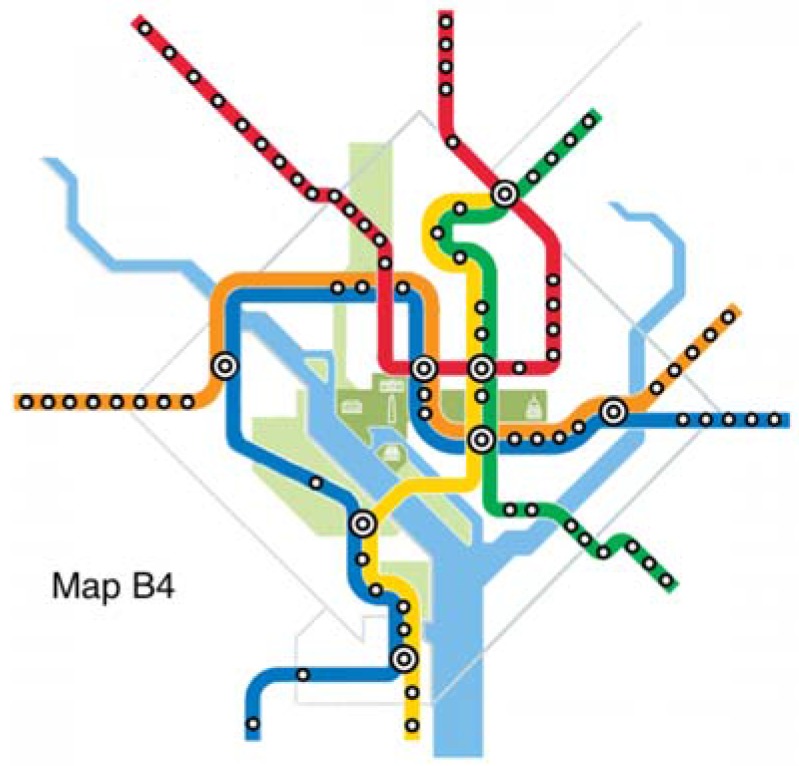
Map B4.

**Figure 7:**
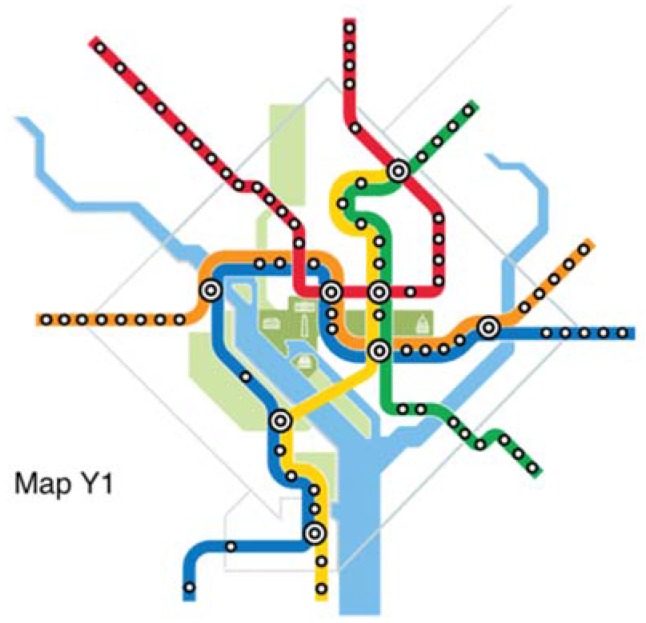
Map Y1.

**Figure 8:**
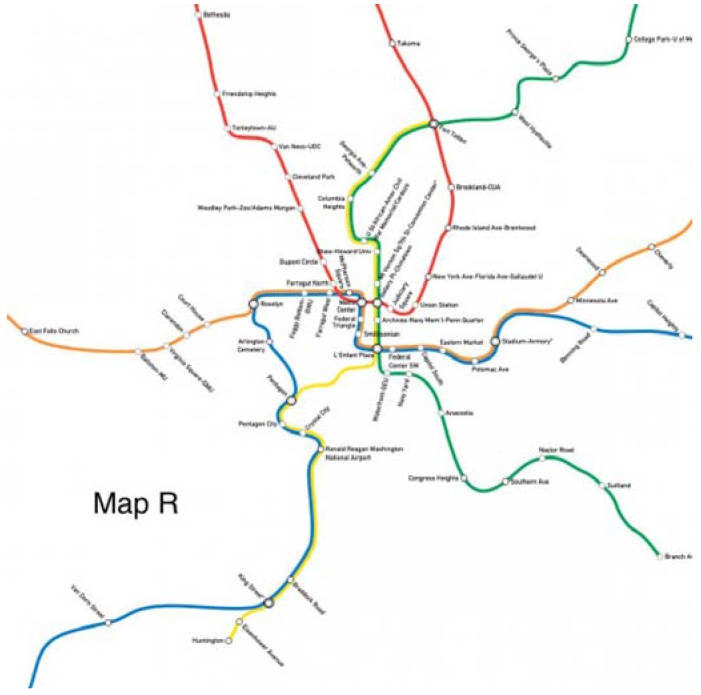
Map R.

The original map and six alternative maps are used in this work (Figure 2, Figure 3, Figure 4, Figure 5, Figure 6, Figure 7 and Figure 8). Map O and R are acquired from Internet open sources, while Map B1–B4 and Y1 follow designs developed in Guo et al. [19] with permission from Guo. Map O is the original schematic map, as shown above and as would have been shown at metro stations in DC. Maps B1 through B4 extend the Blue Line in different directions and lengths. Map Y1 shortens the Yellow Line by streamlining it. Map R is the true map without any distortions. Changes made to create each map are detailed in Table 1 and the alternative maps are shown below in Figure 3, Figure 4, Figure 5, Figure 6, Figure 7 and Figure 8. The Y/B ratio is the ratio of distances between Pentagon and L’Enfant Plaza on the Yellow Line and Blue Line, with unit distance defined as the length of the Yellow Line between the aforementioned stations in the original map, Map O. 

The survey uses thirty of the manually examined Origin-Destination (O-D) pairs developed in Guo et al. [19] with permission from Guo. All selected O-D pairs share the traits of originating due south of Rosslyn in Virginia and of crossing the Potomac River into Washington DC; all present valid trade-offs between a direct but detour route through the Rosslyn Tunnel and a shorter but more indirect (more transfers required) route over the 14th Street Bridge. These 30 O-D pairs combine for 770 daily riders (WMATA *PlanItMetro*).

### 4.2. Survey Procedure

The online route survey, following designs from Guo et al. [19] with permission from Guo, was distributed by the author to several groups of participants in the Washington metropolitan area through several separate means. All subjects gave informed consent for participation before they took part in the study, and were aware of their rights as a voluntary participant. The study was conducted in accordance with the Declaration of Helsinki, and the protocol was approved by the Region 1 Western Massachusetts Scientific Review Committee with a Project Identification Code of 09736-1981540021. 

**Figure 9:**
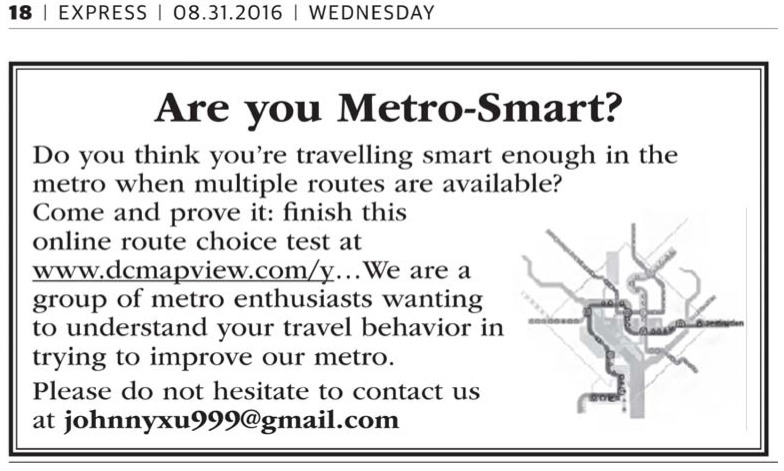
Advertisement as shown on The Express.

Between late 2016 and March 2017, the author designed and posted advertisements titled “Are you Metro Smart?” (Figure 9) for five weeks on The Express to solicit survey responses. The Express is a DC-based, free daily newspaper available at WMATA metro stations, largely targeting daily commuters and usually running out by 9AM. Readers of this free newspaper are primarily frequent riders of the system. 

In December 2016, the author conducted the experiment on Amazon Mechanical Turk (MTurk), an online crowdsourcing platform. MTurk is mostly representative of the general population with regards to gender, race, age, and education [20]. The MTurk experiment restricted the participants to responders from the eight counties covered by the DC Metro service, and the 22 counties in the Washington metropolitan area, paying at $7.5 per hour (beyond the MTurk average rate). Given DC metro’s weaker presence in these regions’ travel markets, most participants through MTurk are presumably moderately or remotely familiar users of the DC Metro system. 

The work, in hoping to test the different behaviors shown by passengers of varying familiarity levels, made initial assumptions about potential responders through The Express and through MTurk that are later validated. Data collected in the survey on usage frequency (Table 2) and participant familiarity (Table 3) show that the various groups are indeed different. 

The survey was supplemented by on-site personal interviews with users. In November 2016, the author traveled to Washington DC to collect public and official opinions on the feasibility of redrawing metro maps to induce behavioral change. 

**Figure 10:**
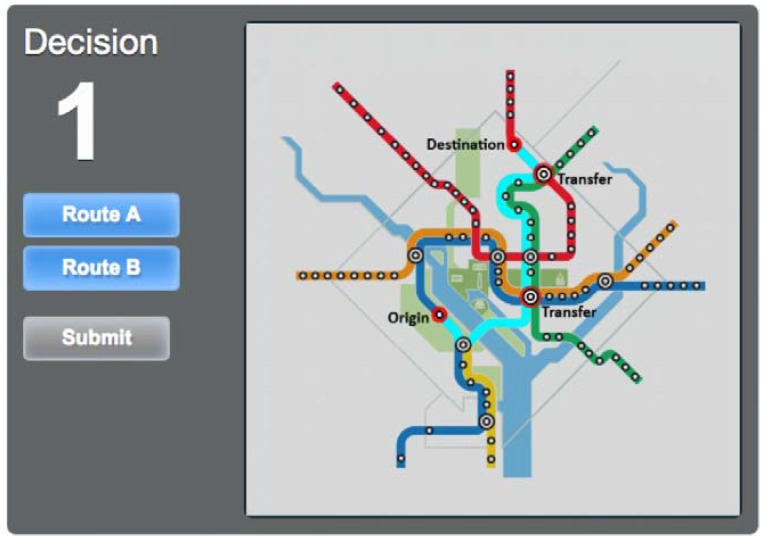
Online survey route choice interface with Route B of an O-D pair highlighted.

With seven maps and 30 Origin-Destination pairs in total, the survey follows the procedures outlined by Guo et al. [19] by randomly presenting each participant one map and eight O-D pairs. For each O-D pair choice, a screen similar to Figure 10 was displayed. By hovering over or tapping the choices, Route A and Route B, participants can see a highlighted route with transfers clearly marked. However, similar to when traveling in the metro itself, no information was given on train frequency, waiting time at the station, or walking distance. Once participants clicked on their desired route and pressed submit, the survey moves on to the next O-D pair on the same map. For this survey, the Silver Line Phase I was disregarded for clarity, as it shares a track with Orange and Blue Lines for the vast majority of its trip and was not relevant to the questions that this paper seeks to answer. 

Two checks were applied to all responses in order to ensure data reliability. First, a trial decision was put in place. Participants were always presented with a specific O-D pair that has one direct route with zero transfers and one roundabout route with two transfers. All participants who chose the latter were excluded from the analysis. The second check is a zip code check. Participant zip codes, one of the basic pieces of demographic information collected by the survey, allow the author to effectively exclude all those residing outside the metropolitan area. After the aforementioned filtering, the advertisement on the Express garnered 204 valid responses and 1632 route choices. The experiment on MTurk received 186 valid responses and 1486 choices.

## 5. Results

It is hypothesized that, in comparison with Map O, the alternative Maps B1–B4 and Y1 would induce more participants to ride on the Yellow Line, and the opposite would be true for Map R. The participants were categorized into three groups: those who responded to advertising on the Express (Passenger), those who responded through MTurk and reported zip codes in one of eight counties directly covered by the WMATA metro service (Resident-8), and those who responded through MTurk reporting zip codes in any of 22 counties in the Washington metropolitan area (Resident-22) (Table 4). Participants in the first group were likely frequent riders when responding, while members in the two resident groups were presumably potential passengers who were less familiar with the metro. The three groups of data presented represent varying degrees of familiarity with the DC metro, covering the spectrum of ridership from most familiar to possibly familiar.

According to the data collected in the experiments, different groups made quite different route choices. Participants in the Passenger group chose the Yellow Line for 72.4% of the trips when shown Map O, our basis for comparison. Maps B1 and B2 induced 79.5% and 81.5% trips to travel through the Yellow Line, 7.1% and 9.1% more than Map O, respectively. These respective differences are significant at the 10% and 5% levels. Maps B3 and B4 performed poorly in comparison, while Map Y1 exhibited a1% significance level (*t* = 2.94), switching 11.2% of riders to the Yellow Line. Lastly, Map R suggests that the current schematic map has already influenced riders to favor the Yellow Line: only 63.8% chose the Yellow Line when Map R was presented, adding an 8.6% ridership to the Blue Line. This real map, Map R, when used or implemented (such as when travelers access actual maps for planning, possibly through online resources), could in fact significantly exacerbate the congestion at Rosslyn. 

## 6. Discussion

### 6.1. Effect of Alternative Maps

Comparing the results among the three groups in Table 4 indicates a vastly interesting pattern. Frequent riders (in the Passenger group) are indeed less responsive to some changes in map design than infrequent riders (in the Resident-8 and Resident-22 groups). For instance, although Map B4 extends the Blue Line length between Pentagon and L’Enfant Plaza by 40%, frequent riders show nearly no signs of behavioral change. The same map leads participants in the Resident-8 and Resident-22 groups to react with 8.8% and 12.1% ridership switches onto the Yellow Line, respectively.

Although participants in the Resident-8 and Resident-22 groups were more responsive to Map B4, frequent riders seem to be more sensitive to other types of map changes that infrequent riders tend to overlook. For example, the 12% decrease in the Yellow Line length in Map Y1 from streamlining does not budge the Resident-8 and Resident-22 groups, but leads to an impressive 11.2% of additional frequent riders choosing the Yellow Line (statistically significant at the 1% level). The real map (Map R) does not ring a bell for the other two groups but influences 8.6% of frequent riders (statistically significant at the 1%) to change their routes. 

A possible explanation is that frequent riders are familiar with Map O, the base scenario. This enables them to pick up salient shape differences on Map Y1 and R and respond accordingly. This familiarity may also visually fool these riders presented one of Maps B1–B4, as these maps follow a similar shape as the base case. 

The large effect on frequent riders from the relatively small change in Map Y1 could be explained by prospect theory [21]. Minimized lengths between Pentagon and L’Enfant Stations (using station positions from Map O) has a Y/B ratio of 0.88/2.7. Map O reflects a 13.6% addition to the minimized Yellow Line length, while Map Y1 brings that length back to minimum. According to prospect theory, changes occurring closer to the reference (in this case, closer to minimum lengths) have more salient effects on utility. Such is the case for familiar riders. For less familiar riders, however, the map presented stands on its own: there is no reference or comparison. For this reason, the resident groups’ behaviors more proportionally reflected Y/B ratios. 

To illustrate the impact of map design on congestion, the number of riders switched can be calculated for the whole metro system. The author hereby presents one case, namely Map Y1 for the Passenger group. For this group, Map O, the benchmark for comparison, led to 556 Yellow Line trips, or 72.2% of all trips on these 30 O-D pairs. Map Y1, on the other hand, had 642 such trips (83.4%). Thus, Map Y1 switched 86 riders from Blue Line onto the Yellow Line, accounting for 11.2% of the daily ridership for these O-D pairs. There are 1452 O-D pairs within the DC metro system with a valid choice between the Rosslyn Tunnel and the 14th Street Bridge. These O-D pairs each involve crossing the Potomac River and all originate or terminate at stations due south of Rosslyn in Virginia, combining for 140,649 daily riders (WMATA *PlanItMetro*). Applying the aforementioned rates of ridership switch (11.2%) to all 1452 similar O-D pairs, Map Y1 switches 15,753 additional riders onto the Yellow Line. This corresponds to 2.6% of the 617,205 daily rides across all 8281 O-D pairs in DC metro (WMATA *PlanItMetro*). Not only are these riders likely daily commuters who would otherwise primarily contribute to peak out crowding, all of this could be done at nearly zero cost and low public disapproval. 

### 6.2. Ethics and Planning

To better understand the public reception of using map design to alter behavior and the true role of schematic maps in passengers’ decision, the author conducted 26 personal interviews in November 2016 at various DC metro stations. 

In responding to a question on how they planned their trips, 88.5% of participants said they use the schematic WMATA map to some extent, including physical maps present at every station or third party apps that have incorporated the official WMATA map, while the remaining 11.5% cited Google Maps (hybrid of schematic and real maps), online trip planners, and hearsay as their primary travel-planning tools. Furthermore, all but five of the participants in the author’s interviews described themselves as commuters to some extent, disproving previous beliefs that frequent riders choose routes entirely based on personal experience. 

When asked if metro maps are accurate representations of reality, an astounding 65.4% of participants believed that the maps presented at metro stations are not schematized *at all* and represent reality as is. This general ignorance among travelers of all levels of familiarity can contribute to the expected low public disapproval of the proposed method. 

When asked whether it is ethical for WMATA to redraw the map to influence their travel choices, 92.3% of the participants believed that this would be acceptable as long as the overall travel time decreases. The overwhelmingly positive reception here may be partly due to the general dissatisfaction among DC metro users towards the metro. However, in a larger sense, this finding effectively clears the public perception hurdle for implementing map design as a planning tool and reaffirms the public belief that positive behavioral nudges are largely acceptable and ethical. 

The author also interviewed planners at WMATA on whether map design will be considered as a possible means to alleviate congestion. Recognizing that the most important purpose of the map is legibility and ease of use for all, planners say that WMATA would “definitely consider” using alternative map designs to solve capacity constraints, calling it a “real possibility” in the upcoming effort to redraw the metro map. The next time WMATA will necessarily revisit the map and consider its redesign will be in 2019 in preparation for opening the Silver Line Phase II in 2020. 

## 7. Conclusions

This paper proposes map design as a possible alternative to large-scale rebuilding or rescheduling to modify traveler behavior and to alleviate congestions. Even minor changes, as this paper shows, can lead large amounts of riders to switch to underutilized lines. Although the DC metro is a special case due to the naturally limited number of options in crossing the Potomac River, and although passengers of varying familiarity levels with the system undoubtedly react differently to different types of changes that the author proposed, the general observations from this work further pave the path for transit maps to begin acting as a low cost tool to mitigate congestion and to alleviate system capacity constraints. Visual nudges such as ones presented in this paper can prove quite powerful in shifting passenger behavior; a more comprehensive set of guidelines on visualization should be drawn in future works to encourage a broader implementation of this relatively new method.

By examining the behavior of familiar passengers, the paper sets the framework for allowing transit maps to affect even the most frequent commuters during peak-hour traffic. This new association contradicts the previously accepted assumption in transportation planning that the familiar passengers would be less responsive to map changes due to their plethora of personal experience. By showing that sensitivity differs from dependency, the paper removes a major obstacle in mitigating congestion through map design. 

Unarguably, the process of redrawing metro maps is one involving public participation as well as aesthetic considerations. Although the author surveyed DC passengers as a means to sample public perception, the line between the appropriate and the inappropriate remains blurred for existing schematic maps. With DC transit passengers overwhelmingly focusing on decreasing overall travel time, the approach outlined can allow passengers to subconsciously satisfy this need, while helping alleviate the system constraints present in the metro. Although surveyed passengers showed enthusiasm for the behavioral nudge, the author believes that excessive public encouragements may hurt the effectiveness of the method’s overall subtle approach, as the proposal relies heavily on unencumbered map sensitivity. 

Similarly, although the author worked with Guo and Wyman in creating alternative maps presented to passengers in the experiments, general aesthetic reception and expectation is hard to gauge. In the following phases of this work, the author seeks to expand the currently limited data set to further investigating the existing patterns and correlations. Some of the insignificant results may not have been due to a lack of association, but to a limited sample size. A factor that begs some future consideration is the different types of transfers available in transit systems. In the DC case, WMATA had recently taken ground-level transfers into practice at two locations. 

Furthermore, DC metro stations include three types of platforms: the side platform, the island platform, and the split platform. The island platform only requires passengers to walk directly across the platform in transferring, making transferring a less demanding task, while the other two types require passengers to change floors as least once. Experienced travelers may take these factors into consideration more than novice users do. 

The notion of schematizing maps to alleviate congestion by taking advantage of passengers’ bounded rationality is a novel one. This paper reaffirms schematic maps’ importance, offers fresh insight into frequent riders’ behavioral malleability, and demonstrates positive public perception towards beneficial manipulations, thus encouraging future literature to formulate a more comprehensive set of guidelines to formalize the means introduced in this paper. Metro maps, given their prominent role in planning and their ability to impact riders during trips, can truly become a thoroughly accepted way to affect traveler behavior and to alleviate crowding. 

## Figures and Tables

**Table 1:** Comparison of map details.

Map	Changed Line	Total Length Changes	Direction Changes	Yellow-Blue Turns	Y/B Ratio
Map O	-	-	-	2-2	1/3
Map B1	Blue	+20%	West	2-1	1/3.6
Map B2	Blue	+20%	North	2-2	1/3.6
Map B3	Blue	+20%	West	2-2	1/3.6
Map B4	Blue	+40%	North + West	2-2	1/4.2
Map Y1	Yellow	−12%	Streamline	1-2	0.88/3
Map R	-	-	-	-	0.96/2.6

**Table 2:** How frequently do you use public transit?

	>5 Days per Week	2–4 Days per Week	Once per Week	1–2 Times per Month	Rarely
Passenger (%)	76.96%	17.65%	2.94%	0.98%	1.47%
Resident-8 (%)	31.52%	11.31%	17.78%	19.19%	20.20%
Resident-22 (%)	23.56%	12.32%	12.85%	20.75%	30.52%

**Table 3:** How familiar are you with the Washington DC metro system?

	Very Familiar	Decently Familiar	Somewhat Familiar	Remotely Familiar	Not Familiar
Passenger (%)	70.10%	23.04%	5.88%	0.98%	0.00%
Resident-8 (%)	57.17%	33.13%	7.27%	0.81%	1.62%
Resident-22 (%)	48.06%	31.06%	12.85%	5.89%	2.14%

**Table 4:** Map effects in three data groups (*** *p* < 0.05, ** *p* < 0.1, * *p* < 0.15).

	Passenger, N = 1632		Resident-8, N = 990		Resident-22, N = 1494	
Map	YL Line (%)	Change & Significance	YL Line (%)	Change & Significance	YL Line (%)	Change & Significance
Map O	72.4%	-	71.6%	-	71.6%	-
Map B1	79.5% **	+7.1%; t = 1.76	77.3%	+5.7%; t = 1.22	77.6% *	+6.0%; t = 1.53
Map B2	81.5% ***	+9.1%; t = 2.32	68.2%	-3.4%; t = 0.57	73.8%	+2.2%; t = 0.50
Map B3	78.4% *	+6.0%; t = 1.55	74.0%	+2.4%; t = 0.44	78.3% *	+6.7%; t = 1.46
Map B4	76.3%	+3.9%; t = 0.96	80.4% **	+8.8%; t = 1.69	83.7% ***	+12.1%; t = 3.00
Map Y1	83.6% ***	+11.2%; t = 2.94	68.2%	−3.4%; t = 0.61	70.7%	−0.9%; t = 0.20
Map R	63.8% ***	−8.6%; t = 1.97	69.6%	−2.0%; t = 0.42	68.1%	−3.5%; t = 0.90
